# The Extraction of Roots—When Is It Indicated?

**Published:** 1872-01

**Authors:** H. L. Sage


### Communications.

### THE EXTRACTION OF ROOTS—WHEN IS IT INDICATED?

BY H. L. SAGE.

A widely spread, if not almost universal opinion seems to prevail with dentists—or at least it is usually borne out by practice—that the root of a tooth, if bereft of its crown, might just as well or had better be removed; that it serves no good purpose, its usefulness being gone; that it is, necessarily, a source of irritation, or that the deleterious effects resulting from its presence in the alveolus more than counterbalances the good derived; in fact, the idea that the extraction of a root, in any instance, is a loss, in any sense of the word, never occurs to many. And why should it, when they do not hesitate to advise, or at least reluctantly (?) consent, to the extraction of whole colonies of teeth, that might just as well be saved by filling, that they may insert their cheap substitutes? While it is true that many, perhaps a majority, of roots of teeth which are presented to our notice, are so diseased, filthy, and noxious that their presence in the mouth is a positive injury, it is equally true, that they may, many times, serve an important purpose, by remaining as sentinels to guard the integrity and prevent the loss of the hard tissues around them, and in other ways be made useful.

The maintenance of the alveoli and the organs implanted therein, is sometimes more dependent on the preservation of roots in a healthful condition than is signified by the usual practice with regard to them. But how seldom is any attempt made to retain them by appropriate treatment. That there *are* times when the retention of the roots of teeth, and the prevention of decay therein, is wise and desirable, it is my purpose to show. Hence the inquiry heading this article.

For example: B. consults Dr. ——— in regard to the feasibility of having an upper partial set of teeth inserted. Most of the teeth back of the cuspidati are yet remaining, while the latter are in good condition. The left central incisor crown is absent, while the root remains. The remaining incisors have cavities in each approximal surface, some of them large, but the majority of medium size. "To make a good thing," he is advised to have all the incisors, including the root, extracted, and a plate with four front teeth inserted. Another, more conservative, would advise the extraction of the root only.

What would be the *true* course here?

First—Fill the cavities in the incisors thoroughly with gold.

Second—Prepare the root for the reception of a gold crown. But if it is in a doubtful condition—dead—even though it has never made trouble, first ascertain if it is in a state to receive it, which may be done by the insertion of a temporary test-filling in the root of floss silk saturated with creosote, and a crown-filling of oxychloride of zinc, or gutta percha. If, after a reasonable time, trouble is experienced, with more or less swelling of the gum, it is obvious, that further treatment must be resorted to before filling with gold.

It may be objected, that a gold crown inserted on a front root would be too conspicuous, disfiguring the individual. Allowing that the objection is a serious one—which I do not, in comparison with the inconvenience and trouble connected with wearing a plate—the root may be prepared for an artificial crown, as detailed in the *Dental Times*, vol. vi, page 36, and thus be protected from decay. But the former method, even, is decidedly preferable to the insertion of a plate of any kind, where natural teeth, or most of them, are remaining; for there is not only some inconvenience attendant upon wearing it, but it has an unfavorable effect upon the remaining teeth, be it ever so nicely adapted to the mouth.

In the act of mastication there is more or less movement of the plate from side to side, which causes a greater or less degree of irritation of the soft parts of the mouth, producing the loss or absorption of the hard tissues, and thus the teeth lose the support therefrom received, often to such an extent that they elongate, loosen, and drop out. A partial plate, with isolated teeth attached, can not be worn without, in this indirect way, causing *some* injury to the adjoining natural teeth.

Again, if it produces irritation of the soft parts, the fluids of the mouth become contaminated or changed in character, and thus act upon the structure of the teeth.

In addition to the above cited objections and inconveniences, the difficulty of speaking is increased, and mastication is less perfectly performed than when the natural organs, or parts of organs, are retained, and the shape and contour of the latter restored. The object should be to avoid wearing a plate, if possible.

Again, suppose, in the above cited case, to carry out the illustration, the root is removed. From this cause, also, the integrity of the arch is impaired; for the loss of the alveolus is, to a certain extent, varying with individuals, soon after, or in time, effected by the process of Nature, who, upon its accomplishment, retires from the particular work in that field of operation, seemingly placing a limit on the duty required of the hard tissues in the immediate vicinity of the root, which was to sustain it in position, dead or alive; but this action having ceased upon its extraction, the adjoining teeth have, by its *removal*, also lost, in a measure, their support.

While there is but one objection to a *well* inserted gold crown on the root of a front tooth—its conspicuousness, but which does not hold good in the case of the back teeth—there are many considerations in its favor, beside those which have already appeared.

To the individual concerned, its presence can not be distinguished from that of the adjoining natural crowns, so far as the sense of feeling, comfort, and efficiency in operation is concerned, while of its permanency and durability for years there can be no question, when the operation is thoroughly and *properly* performed. Noticeable instances of the display of good sense now and then appear, in which the individual, in order to save the remnants of the natural teeth, has quarters, thirds, halves, and wholes of lost crowns replaced with gold, rather than to resort to artificial teeth.

The natural functions are, on the whole, more efficiently aided and conserved by the former course than by the latter.

The following, will afford an example of a case in which the extraction of the roots was *not* indicated. In a mouth, with most of the teeth in good condition, attention was directed to the roots of the left inferior six-year molar, the teeth anterior and posterior thereto being sound. The opposing teeth were also sound and serviceable, and would have been entirely so, had not the superior six-year molar been deprived of its antagonizing tooth; so that all that was needed to utilize the entire machinery of that side was to fill the vacancy caused by the loss of the inferior six-year molar.

The roots had formerly been abscessed, treated, and the pulp and crown cavity filled, but all traces of the operation, excepting, perhaps, a few particles of gold in the extreme portion of the root canal, had ceased to be visible.

The decay extended on the buccal and lingual surfaces, about even with the gum, and below it, on the posterior approximal, or distal, a thin anterior proximal wall only being left standing; so that the roots were deprived of very nearly the entire crown, besides being extensively hollowed out by caries.

By the exercise of patience and very much care, the rubber dam was adjusted, though with difficulty, the root prepared for the reception of the gold foundation—one hundred and four grains of gold, light and heavy—and eleven and one-half hours of time employed, and the operation was eminently successful. During the time allotted to the malleting of the gold—an unbroken period of six hours—not a particle of moisture obtruded upon the filling, and the tooth is now as useful as any of its fellows, and is believed to be so secured, so adapted, so condensed, and so finished as to insure its retention for a long time.

There are other cases in which the extraction of roots is not advisable. Persons whose teeth had presented the characteristics of hardness, density, yellowness; of great firmness, with hard, healthy gums, but which are now worn down, by occlusion, nearly to the margin of the latter, should not permit the extraction of the roots, for it is a question whether they are not, on the whole, capable of greater usefulness than a set of artificial dentures would be. We usually find associated with this class of teeth, thick, bulging alveoli and gums, which render the insertion of *serviceable* dentures quite difficult, and the possessor of the former awkward, if not with little patience in the management of substitutes, especially if *he* has passed the prime of life.

But that the extraction of roots *is* indicated when artificial teeth, with the exception of those on pivots, are to be adapted to the mouth, may, with probable truth, be asserted. In most cases, the irritation produced by the insertion of the plate over roots, would be a sufficient reason for their removal, aside from the fact, that most roots are in such a condition as to be offensive and deleterious. Besides, the permanence and usefulness of the substitute, is compromised and hindered by their presence. In most cases, Nature is constantly at work for the expulsion of roots; and so it is with *entire* teeth which have lost their antagonists; but the former, if mounted with artificial crowns of gold (when the indications exist for such a course), are as likely to be retained as dead natural antagonizing teeth, which are in a state to be tolerated, for they are then in a condition to perform service, the lack of the ability to do which, had previously placed Nature in more formidable antagonism with them, and hence the attempt to expel them from the alveoli.

Again, take a person with teeth, in the main, excellent; who has scrupulous regard to their cleanliness, and is particular in the extreme; with none of the incisors and canines absent; with the crowns of the second superior bicuspids, for instance, gone, but the roots remaining, and not in any manner exerting a deleterious influence in the mouth, and the gums about them healthy.

In such a case, the roots, if sound, may be utilized by building crowns upon them. If this is not done, it is well to fill, at least the pulp cavity, with gold, and thus preserve them, that they may prevent, by their presence, the loss of alveolus.

Many dentists are of the opinion, that incisor and bicuspid crowns of gold can not be rendered permanent; but, having had positive proof to the contrary, I am not disposed to concur with them in this belief. In a case like or similar to the foregoing, the insertion of a plate would seem to be of doubtful expediency, and productive of more injury than benefit.

Even the lateral incisors, which are the smallest teeth in the mouth (if we except the lower central), when decayed to such an extent that the crowns are wanting, may be restored with gold—even the inferior.

If Miss B. makes her appearance with one of the right or left upper or lower lateral incisor roots bereft of its crown, and the other teeth capable of being made useful, it is my duty to advocate such a course, and prepare the root by treatment, if necessary, and the nature of the case will warrant it, for the reception of a gold crown; which, with its *one* objection, of conspicuousness or unnatural appearance, can not overbalance the positive advantages of comfort, natural feeling, ease in speaking, cleanliness, healthfulness, preservation of the hard tissues, the adjoining teeth, etc. This practice may apply, in some cases, not only to one but to several teeth in like condition.

Again, suppose Mr. N., a clergyman, calls. He has an excellent set of natural teeth, in form, structure, firmness, and regular appearance, with a good *physique* to back them. The right superior cuspidatus was filled seventeen years ago, the root included, but three-quarters or more of the crown has, by neglect or otherwise, been suffered to decay and break down. It looks badly and interferes with speech somewhat, which is unfortunate, especially as he is a public speaker. It is a very firm, healthy (though lifeless) root; at least it has no pulp—or periosteum lining the pulp chamber.

What is the course indicated here?

Everything being in readiness, by previous preparation and confidence in its integrity, commence at the end of the root canal, fill, build down, and restore the shape and contour of the tooth. If well done, there is no question about its permanence. Who would advocate extraction in such a case? To do so would be a grievous blunder, to characterize it by no harsher term.

Those who object to contour fillings must confine their objections to fifths, quarters, and thirds of crowns, in which full restoration to shape and size may not be unqualifiedly essential, and then at the risk of being deemed guilty of the folly of choosing the greatest of two evils—always provided the consent of the patient can be obtained to the exercise of the best judgment of the operator.

Here follows the recital of an extreme case. G., a young man of sixteen, calls, with a well developed denture. The second left inferior molar is extensively decayed; the anterior wall is remaining; the buccal has lost one-half of its substance, the lingual three-quarters, the posterior still more, and the gum back of it extends up nearly even with the grinding surface of the tooth. The pulp, being exposed, is destroyed. The rubber dam is adjusted and secured—though, it must be confessed, with difficulty—the cavity is prepared, and, by dint of patience, with other essential virtues and qualifications, the lost portions are successfully duplicated, so far as gold can be made to answer the purpose. In such a case it may be exceedingly difficult to *get at* the *anterior* root, so far as the insertion of gold is concerned. If so, substitute the oxychloride of zinc, and fill over it with the former, making an anchorage in the posterior root, and in other portions by retaining points if necessary. When such an operation has been pronounced an *impossibility*, it is exceedingly gratifying to have been enabled to overcome the more than ordinary difficulties necessarily attendant upon it, and that, too, thoroughly.

Occasionally, wisdom teeth, even, will pay for a good share of such attention, and not only prove that it has not been bestowed in vain, but that the anathemas which have rested upon these tardy accessions to the colony of grinders have been unjustly, or with only partial fairness, hurled against them. If H. has a wisdom tooth which requires from fifty to one hundred grs. of gold, and from six to twelve hours' time to restore it to usefulness, can the expenditure of time, skill, strength and material be counted lost? Not when the results vindicate the wisdom of the undertaking.

We have an illustration of a different class of teeth in the case of Miss C. They are soft and chalky; the enamel is thin, and the cavities are numerous and mostly large. The superior central incisors are abscessed, and will require treatment. After the disease is removed and the roots are filled, one-quarter of the crown will, in each case, require to be restored in shape and contour with gold. Five of the bicuspids will also need attention—presenting cavities for simple and compound fillings. All of the inferior incisors will need approximal fillings. The crowns of the inferior canines are absent, though the roots remain, with healthy but exposed pulps. To insure good results and stability for the fillings, the latter must be extirpated, the roots filled, and the crowns restored to natural shape, contour and size, with gold, and the remaining teeth treated as above indicated; and, with reasonable care and attention on the part of the patient, she may yet receive from them an invaluable amount of service.

There is another class of cavities, in which the decay presents a different appearance from that usually manifested, which, though not exactly coming under the heading of this paper, may yet receive a passing notice, of which the following, may be cited as an extreme but not imaginary case, and as the representative of others similarly, if not so badly affected.

M. has a lateral incisor in which the caries have formed a wide channel entirely around it, with the exception of quite a narrow partition of tooth substance on the lingual or palatine surface. On the labial surface, most of the enamel has been destroyed—a portion, to the extent of about one-quarter of the length of the crown, near and across the cutting edge, only remaining intact—and so in nearly the same proportion with the mesial and distal surfaces, leaving, as nearly as may be described, a hollow cylinder of dentine inclosing the pulp, which, though not quite exposed, has barely escaped. By restoring the lost portions the gold will very nearly encircle the tooth, presenting the appearance of an enamel-pointed gold crown.

Here is a most delicate operation. The retaining points must be made with great care, in order not to impinge upon the pulp; and to avoid fracture, the preparation of the cavity must be conducted with much caution—and for the same reason the malleting of the gold eminently so—while in every step taken, the manipulation must necessarily be characterized by great delicacy of touch. Entire protection from moisture must also be secured, while the gold should be so annealed as to combine the qualities of softness and adhesiveness; the pellets should be small or loosely rolled, and the instruments employed, mostly those with fine points and serrations.

Here, success will crown earnest efforts when guided by skillful hands, as it has before done in cases of a like nature, and thus this lateral incisor placed in a *smiling* condition for years, even though it may have been pronounced *past filling* with anything excepting a second-class material.

> "For of all sad words of tongue or pen,
> The saddest are these: 'It might have been!'"

And with regard to the teeth, people sometimes realize this, after reaping the consequences of indifference, neglect, excesses, and sins of omission and commission. This or that tooth "might have been preserved," has the same sad truth accompanying it, and comes echoing back to many of us with an emphasis which we fully realize, and the personal application of which we shall never cease to regret.

In the presentation of the foregoing examples, the design has been to illustrate a few general principles, which may be applied in practice to many cases of a nature similar to those herein specified.

Is it too much to assert that all roots which can be made useful—without compromising the carrying out of more weighty measures or methods of procedure which promise better results, if such there may be—should be preserved, and if in a healthy condition, maintained therein; or, if not, made so, if practicable; while those with no good purpose to subserve, and whose presence is an injury, should not be allowed to remain? Let us not, in cases over which we have control, sacrifice the greater advantage for the less, or unnecessarily destroy that for the loss of which we can not fully compensate.

The field of conservative dentistry is large, "with ample scope and verge enough" for the full exercise of our limited faculties when engaged in that direction. While it would seem that means, methods and appliances have attained to a degree of efficiency beyond the reach of further progress, we are daily convinced, by the logic of events, that there are yet undeveloped resources from which to recuperate the latent energies of the profession.

